# Challenges in treating children with optic pathway gliomas: an 18-year experience from a middle-income country

**DOI:** 10.3389/fonc.2024.1329729

**Published:** 2024-02-13

**Authors:** Jorge Luis Ramírez-Melo, Daniel C. Moreira, Ana Luisa Orozco-Alvarado, Fernando Sánchez-Zubieta, Regina M. Navarro-Martín del Campo

**Affiliations:** ^1^ Pediatric Oncology and Hematology Service, Hospital Civil de Guadalajara Dr. Juan I. Menchaca, Guadalajara, Mexico; ^2^ Department of Global Pediatric Medicine, St. Jude Children’s Research Hospital, Memphis, TN, United States

**Keywords:** optic pathway glioma, low-grade glioma, global oncology, low-and middle-income countries, visual acuity

## Abstract

**Introduction:**

Patients with optic pathway gliomas (OPG) have good survival rates although their long-term quality of life can be affected by the tumor or treatment-related morbidity. This retrospective study sought to describe the clinical presentation and outcomes of children with OPG at a tertiary center in Mexico.

**Methods:**

Consecutive patients <18 years-of-age with newly diagnosed OPG between January 2002 and December 2020 at the Hospital Civil de Guadalajara Dr. Juan I. Menchaca in Guadalajara, Mexico were included.

**Results:**

Thirty patients were identified with a median age of six years. The most frequent clinical manifestations were loss of visual acuity (40%) and headaches (23%). Neurofibromatosis-1 was found in 23.3% of the patients. Surgery, either biopsy or resection, was done in 20 of 30 patients. Two patients died shortly after initial surgery. The 5-year event-free survival (EFS) was 79.3% ± 10.8% and the 5-year overall survival was 89.5% ± 6.9%. Lower EFS was associated with age less than 3 years, intracranial hypertension at presentation, and diencephalic syndrome. Patients who received surgery as first-line treatment had a 3.1 times greater risk of achieving a performance score of less than 90 points at 6 months after diagnosis (p=0.006). Of 10 patients with vision testing, 5 had improvement in visual acuity, 4 had no changes, and one patient showed worsening.

**Conclusion:**

Our data suggests that favorable outcomes can be achieved with OPG in low- and middle-income countries, although a high rate of surgical complications was described leading to a lower overall survival. These data can be used prospectively to optimize treatment at this institute and other middle-income countries through a comprehensive, multidisciplinary approach.

## Introduction

Central nervous system (CNS) tumors are the second most common cancers in childhood, representing about 20% of cases (1, 2). Among pediatric CNS tumors, 3 to 5% are optic pathway glioma (OPG). OPG are low-grade neoplasms that affect the visual pathway and have a good prognosis, with 5-year overall survival rates frequently reported close to 95% in high-income countries (HICs) (3–5). Nonetheless, OPG have the potential for significant morbidity, from the tumor itself or tumor-directed therapy. Visual deficits and endocrine disturbances frequently affect the quality of life of survivors (6). Poor prognosis has been associated with clinical features, such as young age (less than three years), chiasmatic and hypothalamic invasion, and tumors in patients without neurofibromatosis type 1 (NF-1) (7, 8).

The diagnosis of OPG can usually be made based on neuroimaging and comprehensive clinical examination. Histologic diagnosis is often unnecessary and carries a risk of surgical morbidity, including visual deficits and endocrine dysfunction (9). In patients where treatment is indicated, cytotoxic chemotherapy remains the standard of care, although targeted therapies are becoming more prevalently used as the results of clinical trials are reported (10–13). Radiotherapy can provide better disease control and has better visual outcomes, but due to the long-term side effects, its optimal use remains controversial (14, 15).

About 400.000 children develop cancer worldwide, with 80% residing in low- and middle-income countries (LMICs) (16). The World Health Organization’s (WHO) Global Initiative for Childhood Cancer (GICC) aims to achieve at least a 60% survival for pediatric cancer patients worldwide by 2030 (17). Low-grade glioma (LGG) is one of the six index cancers selected by the GICC to demonstrate the impact of increasing access to quality care for children with cancer (18). Importantly, there are few studies describing the outcomes of the treatment of children with OPG in LMICs (19). The aim of this study was to determine the clinical course and outcomes of children with OPG at the Hospital Civil de Guadalajara Dr. Juan I. Menchaca in Mexico.

## Materials and methods

### Study population

Consecutive patients <18 years-of-age with OPG newly diagnosed between January 2002 and December 2020 treated at the Hospital Civil de Guadalajara Dr. Juan I. Menchaca (HCG) in Guadalajara, Mexico were included. For consideration as an OPG, radiologic characteristics was sufficient, and histologic confirmation was not necessary. The HCG is a regional referral center in the state of Jalisco, on the Pacific coast of Mexico, a middle-income country. The hospital serves approximately 90% of pediatric cancer patients treated with a national health care coverage service in Jalisco. The study was approved by the HCG ethics committee.

### Patient data

Clinical information on demographics, treatment, and follow-up were extracted from institutional medical records. A database was created and included sociodemographic data, NF1 status, pathology, clinical manifestations, tumor location, treatment modalities and timing, performance status, and radiographic response assessment. Data collection was completed in December of 2021.

### Statistical analysis

Quantitative variables were summarized by measures of central tendency statistics (mean or median), while qualitative variables were summarized with absolute frequencies and percentages. Event-free survival (EFS) was defined as the time from diagnosis to first event (progression or death) or for those who were event-free, the date of last contact. For abandonment-sensitive EFS (A-EFS), treatment abandonment was also considered an event. Overall survival (OS) was defined as the time from diagnosis to death or last contact for those still alive. Patients who had not experienced an event by the end of the study were censored at the time of their last follow-up. EFS and OS analyses were performed using the Kaplan-Meier method and compared by the log-rank test (20). Values of p <0.05 were considered statistically significant. SPSS (version 25) was used for analyses.

## Results

### Clinical and demographics characteristics

Thirty patients were included, with a median age of 6 years. Patient characteristics are presented in **Table 1**. The most frequent clinical manifestations were loss of visual acuity (40%) and headaches (23%). Clinical findings consistent with NF-1 were found in 23% of the patients, but genetic confirmation was not available for any of these patients. The criterion for the clinical diagnosis of NF-1 for all patients was the presence of the OPG and at least 6 café-au-lait spots. One child also has a neurofibroma. Nine patients had hydrocephalus at diagnosis. Tissue was obtained in 20 patients (67%), with 2 procedures having non-diagnostic samples.

**Table 1:** Patient’s characteristics, tumor location, and pathology.

Characteristic	Value, n (%)
Sex (n, %)	
*Female*	*19 (63.3)*
**Age (years)**	
*Median (SD)*	*6.0*
Signs and Symptoms	
*Decreased vision*	12 (40%)
*Headache*	7 (23.3%)
*Endocrinopathy*	*5 (16.7%)*
Seizures	4 (13.3%)
*Diencephalic syndrome*	5 (16.6%)
*Proptosis*	3 (10%)
Neurofibromatosis	
*No*	23 (76.6%)
*Yes*	7 (23.3%)
Tumor location	
*Optic nerve*	6 (20%)
*Optic nerve/chiasmatic*	4 (13.3%)
*Chiasmatic*	3 (10%)
*Chiasmatic/Hypothalamic*	15 (50%)
*Chiasmatic/Hypothalamic/optics tracts*	2 (6.6%)
Histology	
*No tissue obtained*	10 (33. 3%)
*WHO Grade 1*	16 (53.3%)
*WHO Grade 2*	2 (6.6%)
*Reactive gliosis*	2 (6.6%)

### Surgical approach and outcomes

Neurosurgical procedures were performed in 20 patients: tumor biopsy for 11 patients and resections in 9 cases. None of these patients were evaluated by pediatric oncology before surgery. The acute complications associated with these surgical procedures included two deaths (2/20, 10%): one due to catecholamine-resistant shock, and one patient with meningitis after surgery, with subsequent shunt failure and septic shock. The patients who died from acute complications related to surgery occurred in 2006 and 2008. Additional features of the patients for whom biopsy or resection was performed are included in **Table 2**.

**Table 2:** Surgical outcomes.

Patient	Localization	Surgical indication	Degree of resection	Postsurgical complications
1	Chiasmatic, hypothalamic	Intracranial hypertension	NTR	Cerebrospinal fluid fistula, subdural hematoma
2	Optic nerve	Unclear	NTR	Ocular hematoma
3	Chiasmatic, hypothalamic	Differential diagnosis with craniopharyngioma	NTR	Bilateral subdural hematomas, left facial paralysis, left nasal heteronymous hemianopsia, quadriparesis, worse functional status
4	Chiasmatic, hypothalamic	Unclear	NTR	Left facial palsy, unilateral left nasal hemianopsia
5	Chiasmatic, hypothalamic	Intracranial hypertension	NTR	Hypogonadism, hypothyroidism, worse functional status
6	Chiasmatic, hypothalamic	Intracranial hypertension	NTR	None
7	Chiasmatic	Unclear	STR	Catecholamine-resistant shock and death
8	Chiasmatic and hypothalamic	Unclear	STR	Hemiplegia and diabetes insipidus
9	Chiasmatic	Intracranial hypertension	STR	None
10	Chiasmatic, hypothalamic, and optical radiation	Intracranial hypertension	Biopsy	Neurological infection, shunt failure, death due to septic shock
11	Optic nerve and chiasmatic	Unclear	Biopsy	Transfusion for acute bleeding
12	Chiasmatic and hypothalamic	Intracranial hypertension	Biopsy	Neurological infection and intra-abdominal abscess
13	Chiasmatic	Unclear	Biopsy	None
14	Chiasmatic, hypothalamic	Intracranial hypertension	Biopsy	Pneumothorax, cardiorespiratory arrest, wound dehiscence, bone defect, neurological infection, shunt failure.
15	Optic nerve	Unclear	Biopsy	None
16	Chiasmatic, hypothalamic, and optical radiation	Unclear	Biopsy	None
17	Chiasmatic and hypothalamic	Unclear	Biopsy	Decreased visual acuity
18	Optic nerve	Unclear	Biopsy	None
19	Chiasmatic and hypothalamic	Intracranial hypertension	Biopsy	Neurological infection, valvular dysfunction
20	Chiasmatic, hypothalamic	Intracranial hypertension	Biopsy	None

### Adjuvant treatment and outcomes

**Figure 1:**
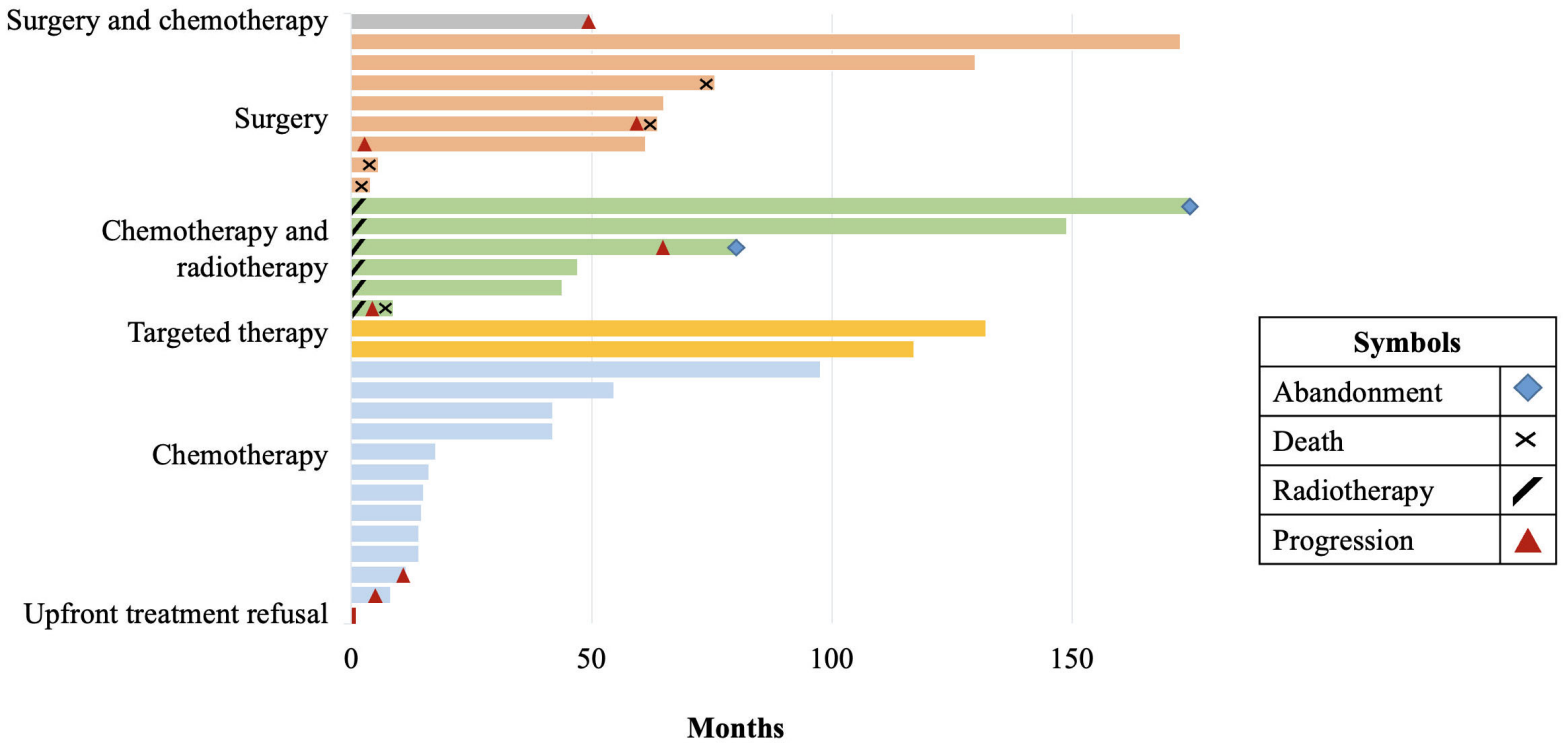
Swimmer’s plot of cohort.

Overall, 12 patients (40%) received chemotherapy, all of them with carboplatin: seven as monotherapy and 5 in combination with vincristine. Hypersensitivity to carboplatin was presented in 28.5% of the patients. No severe cases were reported, and patients were able to continue treatment with carboplatin. Three patients started active surveillance at diagnosis because they had no evidence of visual deficit, but after 3, 4, and 5 months respectively visual changes were found, and chemotherapy treatment was started. Two patients had tumor progression while receiving chemotherapy as first-line treatment. Both patients presented progressive disease while receiving carboplatin treatment and therapy was changed to weekly vinblastine with stable disease until the last follow-up. The treatment and outcomes of the 30 patients are included in **Figure 1**.

Two patients with NF-1 received targeted therapy with sirolimus as first-line treatment. Both patients were asymptomatic at the last follow-up and had stable disease. The combination of chemotherapy and radiotherapy was used as initial treatment in 6 patients (20%). Two patients with unresectable suprasellar tumors, three children with an unresectable thalamic/hypothalamic tumor with quick clinical deterioration, and one patient treated before 2005 without clear indication. Three patients who received chemotherapy and radiotherapy were treated with a chemotherapy regimen based on carmustine, vincristine, and prednisone, all treated before 2007. One patient had surgery and chemotherapy as first-line treatment due to extensive residual tumor.

### Survival outcomes

The median follow-up was 5 years (0.3-14.1 years). Three patients abandoned treatment (10%). One patient denied upfront treatment, another patient abandoned the treatment after tumor progression, and one case due to unknown reasons. Five patients died. Two deaths were related to surgery as mentioned above. One death was due to severe post-surgery neurological sequela and aspiration pneumonitis in the sixth year of follow-up. One patient had tumor progression and subsequently died due to *pneumocystis* pneumonia. Finally, one patient with an extensive hypothalamic tumor died due to sodium imbalance and pontine myelinolysis.

**Figure 2:**
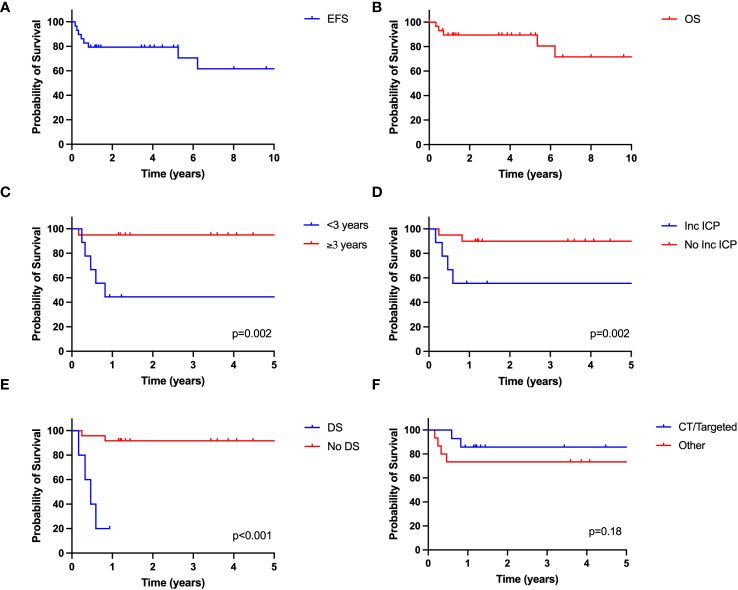
Outcome of pediatric OPG in Mexico. **(A)** EFS; **(B)** OS; **(C)** EFS based on age; **(D)** EFS based on presence of increased intracranial pressure (ICP) at presentation; **(E)** EFS based presence of diencephalic syndrome (DS); **(F)** EFS based on treatment. CT, chemotherapy.

The 5-year EFS was 79.3% ± 10.8% (**Figure 2A**) and the 5-year OS was 89.5% ± 6.9% (**Figure 2B**). The 10-year EFS was 61.7% ± 19.1% and the 10-year OS was 71.6 ± 16.8%. The 5-year A-EFS 76.7% ± 11.4% and the 10-year A-EFS was 59.6 ± 19.2%. Lower EFS was associated with age less than 3 years (**Figure 2C**), intracranial hypertension (**Figure 2D**), diencephalic syndrome (**Figure 2E**). The first-line treatment used was not associated with EFS (**Figure 2F**).

### Functional outcomes

Ten of 30 patients had a visual assessment available before and after treatment. Five patients had improvement in visual acuity, 4 patients had no changes in visual acuity, and one patient showed worsening (**Table 3**). At 6 months from diagnosis, the Lansky/Karnofsky performance score was higher in patients who received chemotherapy or targeted therapy compared to those who had a surgical resection. The patients who received surgery as part of first-line treatment had a 3.1 times greater risk of having a Lansky/Karnofsky performance score lower than 90 points (p = 0.006).

**Table 3:** Clinical features and visual outcomes.

Patient	Age	Location and treatment	Test	Baseline	Response
1	4 years	Right ON, Chemotherapy	logMAR visual acuity	OD: counting fingers, 0.5 meters OS: 0.3	OD: counting fingers 1 meters OS: 0.3
			Visual field	Not done	OD: Paracentral scotoma LE: Normal
			Visual potentials	Demyelination and axonal deficit type visual pathway disorder	Not done
			Lansky/Karnofsky	90	90
2	11 years	Right ON, Chemotherapy	logMAR visual acuity	OD: 1 OS: 0.0	OD: 0.0 OS: 0.0
			Lansky/Karnofsky	90	100
3	14 years	Chiasm and hypothalamus, Chemotherapy	logMAR visual acuity	OD: VFI 3% OS: VFI 5%	OD: VFI57% OS: VFI31%
			Lansky/Karnofsky	90	100
4	7 years	Left ON Chemotherapy	logMAR visual acuity	OD: 0.1 OS: no light perception	OD: 0.1 OS: no light perception
			Lansky/Karnofsky	90	90
5	3 months	Chiasm and hypothalamus, Chemotherapy	logMAR visual acuity	OD: fixes and follows a target OS: fixes and follows a target	OD: fixes and follows a target OS: fixes and follows a target
			Visual potentials	Bilateral and symmetric lesion, more severe in LE	Normal
			Lansky/Karnofsky	100	100
6	8 years	Right ON Chemotherapy	logMAR visual acuity	OD: 0.1 OS: 0.0	OD: 0.0 OS: 0.0
			Visual field	OD: VFI76% OS: VFI 92%	OD: VFI96% OS: VFI99%
			Lansky/Karnofsky	90	100
7	4 years	Chiasm and hypothalamus, Chemotherapy	logMAR visual acuity	OD: counting fingers 2 meters OS: no light perception	OD: counting fingers 2 meters OS: no light perception
			Lansky/Karnofsky	60	100
8	6 months	Chiasm and hypothalamus, Chemotherapy	logMAR visual acuity	OD: fixes, follows, and keeps their vision focused on a target OS: fixes, follows, and keeps their vision focused on a target	OD: fixes, follows, and keeps their vision focused on a target OS: fixes, follows, and keeps their vision focused on a target
			Visual potentials	Bilateral and symmetric lesion, more severe in OS	Normal
			Lansky/Karnofsky	100	100
9	7 years	Bilateral and Chiasm, Sirolimus	logMAR visual acuity	OD: 0.3 OS: 0.3	OD: 0.3 OS: 0.3
			Lansky/Karnofsky	90	100
10	9 years	Chiasm and hypothalamus, Surgery	logMAR visual acuity	OD: 0.17 OS: 0.3	OD: 0.3 OS: 0.3
			Lansky/Karnofsky	90	70

ON, Optic Nerve; OD, oculus dexter; OS, oculus sinister; VFI, Visual Field Index.

## Discussion

This study of patients with OPG treated in Mexico allows for the evaluation of multiple elements of the care of children with these tumors and their outcomes. Our data suggests that favorable outcomes can be achieved with OPG in LMICs, although these are still lower than HICs. Factors such as young age, intracranial hypertension, and diencephalic syndrome continue to portend prognostic significance.

Although LGG are the most common CNS tumor in children, the global burden of LGG is unknown. The comprehensive evaluation of pediatric cancer incidence and mortality rates relies on quality population-based cancer registries (21). Important for LGG, and specifically for OPG, tumors that are not histologically confirmed are inconsistently captured in cancer registries (22). Furthermore, the International Classification of Childhood Cancer, does not segregate pediatric CNS tumors into many clinically relevant groups, such as LGG (23). These factors lead to a limited understanding of outcomes LGGs across the world, making peer-review publications key in describing survival rates.

Consistent with reports from LMICs (10), our study showed inferior outcomes for children with OPG compared to HICs, specifically when considering OS (4, 5). Worse outcomes were influenced by a high rate of post-operative complications. In our study, the complications presented by patients who received surgical resection at diagnosis were frequent and included two deaths. Although surgery can be curative for pediatric low-grade gliomas in other locations, resection of OPG is rarely indicated (10). Furthermore, patients with resection had a greater risk of achieving lower performance scores. As mentioned, none of the patients who had resections were seen by pediatric oncology prior to the surgery. This rate of complications clearly exemplifies the need for comprehensive, multidisciplinary care for children with OPGs starting at the time of diagnosis. A pediatric neuro-oncology program now exists at the HCG, so it is hoped that outcomes for all children with CNS treated at the institution will improve.

Systemic chemotherapy is usually considered the first-line treatment for OPG due to the risk of complications of radiotherapy (10). In our cohort, 20% patients received both radiotherapy and chemotherapy as front-line therapy. This points to an overuse of radiotherapy and the likelihood of an increased burden of long-term morbidity for these patients. Although we sought to evaluate long-term functional outcomes, comprehensive neuro-cognitive testing was not available for the patients of this cohort and other complications of radiotherapy were not captured. Further studies are needed to evaluate if radiotherapy is more prevalently used for OPG in LMICs and what factors could lead to this phenomenon.

In the last years, in HICs, targeted therapies for LGG are being used more frequently to achieve disease control (10). In our study, sirolimus was used in two patients with NF-1 and visual impairment, as this medication is more accessible and less costly than BRAF or MEK inhibitors. The use of mTOR inhibitors has been reported for patients with LGG (11, 24, 25), but may warrant further investigation in contexts where BRAF or MEK inhibitors are not available for patients. Among the challenges for pediatric cancer care in LMICs is the availability and affordability of chemotherapy (26). Although drugs like vincristine, carboplatin, and vinblastine are on the WHO essential medicines list, targeted agents relevant for the care of OPG, like MEK inhibitors, are not included (27). These agents could be relevant in LMICs as they have no impact on patient immunity and hence, decrease the need of hospital admissions due to acute complications. Advocacy efforts to increase access to novel agents is imperative to the care of children with LGG across the world. Importantly, a greater use of targeted therapy must align with increase access to the molecular diagnostics needed to identify the patients who would benefit from targeted therapy.

In evaluating the response to treatment of optic pathway gliomas, preservation of visual function is a key goal of treatment. In this series, the most frequent presenting sign was decreased visual acuity and the visual outcomes of 10 patients was included, with improvement in a subset of patients. Data on functional outcomes of pediatric LGG in LMICs are exceedingly sparse (19). Functional outcomes be investigated more in depth in LMICs as this is a key parameter of outcomes for this disease.

We report an abandonment rate of 10%. Treatment abandonment is a complex phenomenon associated with social, economic, and treatment-related factors (28). Importantly, universal healthcare existed for children and adolescents with cancer in Mexico after 2004, timeframe when most of this cohort was treated (29, 30). Additional analyses, including social, economic, and treatment-related factors, are necessary to identify those associated with an increased risk of treatment abandonment for patients with LGG.

This study has limitations to be mentioned. As a retrospective study, all details of diagnosis and treatment was not available for some patients, especially as we sought to extract detailed features of care and outcomes. In addition, although some functional outcomes were included, more robust parameters would be needed to describe the burden of disease in these patients and the impact on quality of life. Furthermore, visual status and follow-up tests was only available in a subset of patients.

The treatment of patients with OPG is focused not only survival but improving the quality of life from both the disease and treatment. The comprehensive, multidisciplinary care of patients during their disease is essential to define the optimal treatment options. Building a robust understanding of the care that exists for patients with OPG in LMICs is needed to define interventions that would lead to improved quality of care for these patients.

## Data availability statement

The raw data supporting the conclusions of this article will be made available by the authors, without undue reservation.

## Ethics statement

The studies involving humans were approved by Hospital Civil de Guadalajara ethics committee/institutional review board. The studies were conducted in accordance with the local legislation and institutional requirements. The ethics committee/institutional review board waived the requirement of written informed consent for participation from the participants or the participants’ legal guardians/next of kin because Written informed consent was waived as this was a retrospective study.

## Author contributions

JR: Formal analysis, Visualization, Writing – review & editing, Conceptualization, Data curation, Investigation, Writing – original draft. DM: Formal analysis, Methodology, Writing – review & editing, Visualization. AO: Investigation, Writing – review & editing. FS: Investigation, Writing – review & editing. RN: Investigation, Writing – review & editing, Conceptualization, Data curation, Formal analysis, Methodology, Resources, Supervision, Validation.
